# Profiling Gene Expression Induced by Protease-Activated Receptor 2 (PAR2) Activation in Human Kidney Cells

**DOI:** 10.1371/journal.pone.0013809

**Published:** 2010-11-02

**Authors:** Jacky Y. Suen, Brooke Gardiner, Sean Grimmond, David P. Fairlie

**Affiliations:** Institute for Molecular Bioscience, The University of Queensland, Brisbane, Queensland, Australia; National Cancer Institute at Frederick, United States of America

## Abstract

Protease-Activated Receptor-2 (PAR2) has been implicated through genetic knockout mice with cytokine regulation and arthritis development. Many studies have associated PAR2 with inflammatory conditions (arthritis, airways inflammation, IBD) and key events in tumor progression (angiogenesis, metastasis), but they have relied heavily on the use of single agonists to identify physiological roles for PAR2. However such probes are now known not to be highly selective for PAR2, and thus precisely what PAR2 does and what mechanisms of downstream regulation are truly affected remain obscure. Effects of PAR2 activation on gene expression in Human Embryonic Kidney cells (HEK293), a commonly studied cell line in PAR2 research, were investigated here by comparing 19,000 human genes for intersecting up- or down-regulation by both trypsin (an endogenous protease that activates PAR2) and a PAR2 activating hexapeptide (2f-LIGRLO-NH_2_). Among 2,500 human genes regulated similarly by both agonists, there were clear associations between PAR2 activation and cellular metabolism (1,000 genes), the cell cycle, the MAPK pathway, HDAC and sirtuin enzymes, inflammatory cytokines, and anti-complement function. PAR-2 activation up-regulated four genes more than 5 fold (DUSP6, WWOX, AREG, SERPINB2) and down-regulated another six genes more than 3 fold (TXNIP, RARG, ITGB4, CTSD, MSC and TM4SF15). Both PAR2 and PAR1 activation resulted in up-regulated expression of several genes (CD44, FOSL1, TNFRSF12A, RAB3A, COPEB, CORO1C, THBS1, SDC4) known to be important in cancer. This is the first widespread profiling of specific activation of PAR2 and provides a valuable platform for better understanding key mechanistic roles of PAR2 in human physiology. Results clearly support the development of both antagonists and agonists of human PAR2 as potential disease modifying therapeutic agents.

## Introduction

Currently ∼900 human G protein-coupled receptors (GPCRs) are annotated, forming a diverse family of membrane-spanning cell-surface proteins that may account for >2% of the human genome [1], [2]. Typically GPCRs are single polypeptide chains containing seven membrane-localized helices connected by three extracellular and three intracellular loops, with extracellular amino and intracellular carboxyl termini. Both extracellular and intracellular domains vary substantially in size, the former having evolved to selectively recognize many types of GPCR-activating extracellular ligands, while the latter mediate signal transduction through coupling to combinations of G proteins resulting in extensive functional diversity [3]. Protease activated receptors (PARs) are unusual GPCRs [4] with as yet no known endogenous extracellular ligands. PARs are however indirectly activated by proteases which cleave the N-terminus of at least four PAR isoforms, exposing a new N-terminus that folds back and intramolecularly self-activates PAR [5]. Short synthetic peptides corresponding to the new N-terminus can trigger PAR activation, but only at much higher concentrations than proteases [4]. The most active reported PAR2 agonist is the hexapeptide 2-furoyl-LIGRLO-NH_2_ (EC_50_∼200 nM). PAR2 is activated by mainly serine proteases (e.g. trypsin, tryptase, cathepsin G) but not thrombin and may be linked to inflammatory and proliferative disorders [4].

PAR2 activation has been linked to cancer progression, especially metastasis and angiogenesis [6], [7], [8], [9], as well as pro-inflammatory [10], [11], [12], [13], [14], [15] and anti-inflammatory [16], [17] properties depending on the system, although this is controversial and not well understood. PAR2 activation reportedly causes blood vessel relaxation, increased vascular permeability, leukocyte adhesion [18], and release of pro-inflammatory cytokines (e.g. IL-1β, IL-6, IL-8, TNF-α) and Intracellular Cell Adhesion Molecules-1 (ICAM-1) from human blood monocytes [19], [20]. PAR2 deficient mice show impaired production of IgE and IL-4 [21], reduced contact sensitivity in a model of allergic inflammation in the airways [22], and resistance to adjuvant-induced arthritis [12] or delayed onset of inflammation [23]. PAR2 is reportedly implicated in the pathogenesis of cardiovascular disease [24], gastric ulcers [25], [26], asthma [15], [27], and liver fibrosis [28], [29].

On a cautionary note, many cellular and physiological effects of PAR2 activation have been implicated solely through the use of PAR2 peptide agonists (e.g. SLIGKV-NH_2_, SLIGRL-NH_2_, 2-furoyl-LIGRLO-NH_2_) now known to be non-selective for PAR2 over other targets, even though selective over PAR1. Such agonists can activate neurokinin-1 receptor and other receptors [30], [31], [32], [33]. The only known antagonist of trypsin-induced PAR2 activation, reportedly an inhibitor of TNF-α and IL-1β release [34], has only mM affinity for PAR2 and selectivity for this one receptor is extremely unlikely. Our understanding of PAR2 in human physiology and disease is thus still limited by the lack of truly selective and potent ligands suitable for *in vivo* studies.

For these reasons we have used a microarray approach, with two structurally and mechanistically different PAR2 agonists, to clearly establish effects of PAR2 activation on human gene expression. We compare intersecting gene expression profiles following separate PAR2 activation by a peptide (2f-LIGRLO-NH_2_) versus a serine protease (trypsin), expecting that genes up- or down- regulated by both agents might help identify cellular pathways associated with PAR2 activation. We studied human embryonic kidney cells HEK293 because of their widespread use in PAR2 research [4], their presence on kidney epithelial, mesangial, and infiltrating renal inflammatory cells [35], [36], [37], known Ca^2+^ release from HEK293 cells treated with trypsin and PAR2 activating peptides [38], [39], and because of possible roles for PAR2 in inflammation, ion transport, blood flow regulation, cell growth and repair in the kidney [37]. A limited gene expression profile has been reported for PAR1 activation by thrombin using cDNA microarray analysis, with 65 genes induced in HMEC-1 cells [40], enabling some comparison between PAR2 and PAR1 activation on gene expression. Here we show that PAR2 activation in HEK293 cells impacts widely on metabolic and proliferative cell cycle pathways, and on some notable mediators of the inflammatory immune response.

## Results

### Genes regulated by trypsin and peptide agonists

**Figure 1:**
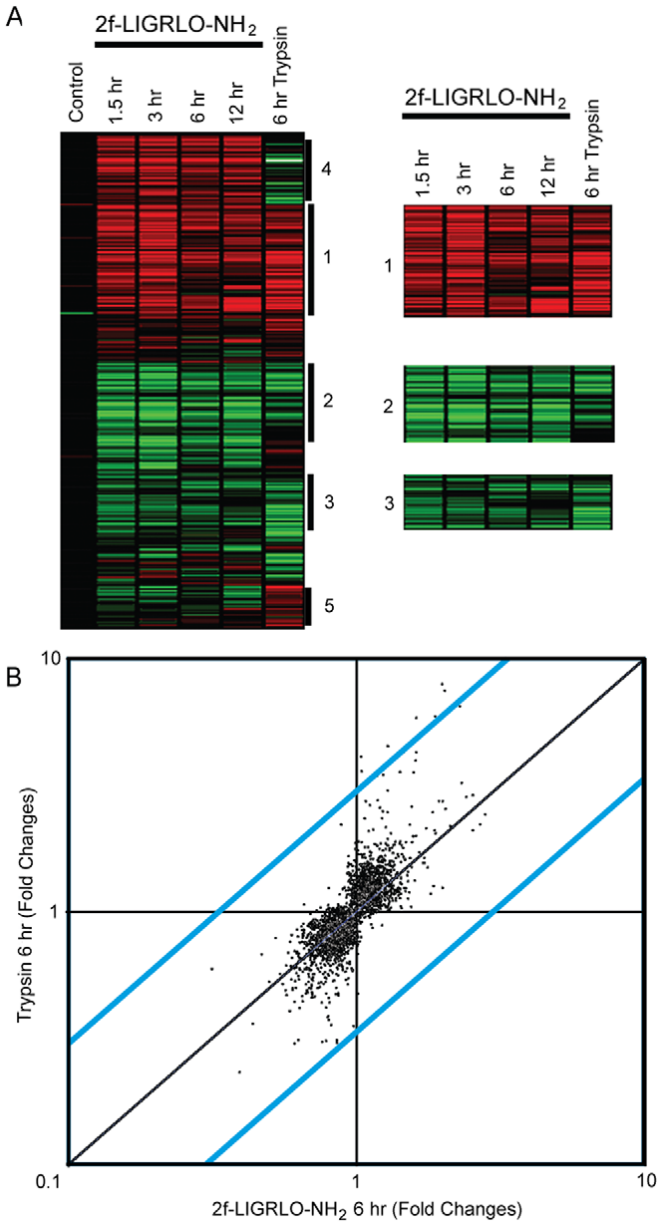
Microarray analysis of PAR2 induced gene expression. HEK293 cells were treated with either trypsin (50 nM) or 2f-LIGRLO-NH_2_ (1 µM). Total RNA was harvested at different time points after treatment (Trypsin, 6 h; Agonist, 1.5, 3, 6, 12 h). Control and treated cDNAs were hybridized to the ∼19,000 cDNA microchip and results were quantified, normalized and filtered as outlined in experimental procedures. (A) Heatmap (red, up-regulated; green, down-regulated) featuring 5 gene clusters. Three clusters show similar modulation by PAR2 peptide agonist and trypsin (1, 2, 3). Cluster 1 shows genes up-regulated by PAR2 activation, clusters 2 and 3 show genes suppressed by PAR2. Clusters 4 and 5 show opposing effects resulting from PAR2 peptide agonist versus trypsin. (B) Scatter plot of genes regulated by trypsin and PAR2 agonist peptide after 6 h treatment. Dots in right hand top quadrant are genes up-regulated by both trypsin and peptide, those in bottom left hand quadrant are genes down-regulated by both treatments. Blue lines depict regions where gene expression increased or decreased by≤3 fold.

To identify candidate genes that might be differentially expressed as a result of PAR2 activation in human embryonic kidney cells (HEK293), cDNA microarray analyses were performed using a human Compugen array of ∼19,000 different human genes. HEK293 cultures were treated with a high dose of either PAR2 activating peptide (2f-LIGRLO-NH_2_, 1 µM) or trypsin (50 nM) for 1.5–12 h. Total RNA was then isolated from treated versus untreated cells at 1.5, 3, 6, 12 h following PAR2 activation. Control and treated cDNA were hybridized to array chips simultaneously. Raw data was quantified, normalized and filtered according to criteria above. There were 5,270 genes that changed significantly (P<0.01) in response to either trypsin or 2f-LIGRLO-NH_2_. These genes were categorized into 5 clusters based on whether upregulated (cluster1) or downregulated (clusters 2 and 3) by both treatments (Figure 1), upregulated by PAR2 activating peptide but downregulated by trypsin (cluster 4), or downregulated by PAR2 activating peptide but upregulated by trypsin (cluster 5). About half of these genes were similarly up- or down- regulated by both treatments (clusters 1–3).

The objective was to focus on identifying an intersecting set of genes that were either up- or down- regulated significantly by both methods of PAR2 activation. This approach was taken because trypsin is highly promiscuous in its effects on cell surface proteins, and the agonist peptide (2f-LIGRLO-NH_2_) has been shown to be selective for PAR2 over PAR1 [11] but little information is available about its selectivity for PAR2 over other GPCRs or other receptors. Therefore we considered that results obtained from either treatment alone would not clearly define specific gene expression profiles that could be unambiguously attributed to PAR2 activation. However, by comparing genes regulated in a similar fashion (both up- or both down-) by both treatments, it seemed likely that the common effects could be directly attributed to activation of their common receptor PAR2, since the protease and short peptide are unlikely to share other mechanisms.

### Key genes regulated by PAR2 activation

Expression of PAR2 mRNA itself was found to slightly increase upon treatment with trypsin (1.7 fold) or PAR2-AP (1.2 fold) for up to 6 h. The effects eventually returned to baseline 12 h after stimulation, but were significant enough to qualify as evidence that PAR2 expression was upregulated in response to PAR2 agonists.

A number of genes were upregulated more than 4 fold or downregulated more than 3 fold in response to both PAR2 agonists. The two most up-regulated genes were dual specificity protein phosphatase 6 (DUSP6) and WW domain-containing oxidoreductase (WWOX) with 7.5 fold and 8 fold increases respectively. Others were AREG, SERPINB2, IL-8, THUMPD1, and SPRY2. Also there were 6 genes that were 3 fold or more down-regulated versus normal (e.g. 0.3 fold of control). These genes are TXNIP, RARG, ITGB4, CTSD, MSC and TM4SF15. More detailed profiles of these genes are in Table 1 and discussed ahead.

**Table 1:** Genes regulated most strongly via PAR2 activation by either 2f-LIGRLO-NH_2_ (2f-O) or trypsin.

Name	Gene	2f-O^2^ 1.5h	2f-O 3h	2f-O 6h	2f-O 12h	Trypsin^3^ 6h
*Up-Regulation*						
Dual Specificity phosphate 6	DUSP6	2.3^1^	2.6	2.0	1.4	7.5
WW-domain-containing oxidoreductase	WWOX	5.5	1.5	2.0	0.7	8
Amphiregulin preproprotein	AREG	2.5	2.9	1.6	1.3	5.8
Serine proteinase inhibitor, clade B, member 2	SERPINB2	1.6	2.5	2.3	1.3	6.5
Interleukin-8	IL-8	2.4	1.9	1.3	2.8	4.5
THUMP domain containing 1	THUMPD1	4.3	2.4	2.8	1.9	2.4
Sprouty homolog 2	SPRY2	2.0	2.0	1.4	1.4	3.6
*Down-Regulation*						
Thjoredoxin interacting protein	TXNIP	0.3	0.6	0.6	0.7	0.5
Retinoic acid receptor, gamma	RARG	0.6	0.4	0.8	0.7	0.3
Integrin, beta 4	ITGB4	0.6	0.6	0.9	0.8	0.3
Cathepsin D	CTSD	0.7	0.6	0.9	0.7	0.3
Musculin	MSC	0.5	0.6	0.8	0.7	0.3
tetraspanin 15	TM4SF15	0.5	0.3	0.7	0.7	0.9

*^1^Expression level expressed as fold differences of control (control  = 1.0)*.

*^2^2f-O: 2f-LIGRLO-NH_2_ 1 µM*.

*^3^Trypsin: Human Trypsin 50 nM*.

### PAR2 activation and the cell cycle

PAR2 has been linked to cellular proliferation and cancer biology. Microarray data were therefore checked for these cellular functions and 136 genes associated with cell cycle regulation were found to be affected similarly by both trypsin and agonist peptide. A representative group of 55 such genes are listed in Tables 2 and 3.

**Table 2:** Cell cycle genes down-regulated^1^ through PAR2 activation by either 2f-LIGRLO-NH_2_ or trypsin.

Name	Gene	2f-O^2^ 1.5h	2f-O 3h	2f-O 6h	2f-O 12h	Trypsin^3^ 6h
B-cell CLL/lymphoma 3	BCL3	0.6	0.8	0.9	0.9	0.7
cyclin F	CCNF	0.7	0.7	0.9	0.8	0.9
cell division cycle 20 homolog	CDC20	0.7	0.7	0.9	0.8	0.9
cyclin-dependent kinase (CDC2-like) 10	CDK10	0.5	0.4	0.7	0.6	0.8
cyclin-dependent kinase 5	CDK5	0.8	0.8	0.9	1.0	0.7
chromatin licensing and DNA replication factor 1	CDT1	0.7	0.7	0.8	0.8	0.8
c-src tyrosine kinase	CSK	0.6	0.5	0.8	0.7	0.9
mitogen-activated protein kinase 3	ERK1	0.7	0.7	0.8	0.7	0.9
forkhead box O4	FOXO4	0.8	0.7	0.9	0.8	0.6
G protein pathway suppressor 2	GPS2	0.6	0.5	0.8	0.6	0.9
histone deacetylase 3	HDAC3	0.7	0.6	0.8	0.7	0.8
histone deacetylase 7A	HDAC7A	0.5	0.4	0.7	0.6	0.9
inhibitor of growth family, member 4	ING4	0.6	0.6	0.7	0.8	0.7
inhibin, alpha	INHA	0.8	0.7	0.8	0.7	0.5
jagged 2	JAG2	0.6	0.6	0.7	0.7	0.8
latent transforming growth factor beta binding protein 2	LTBP2	0.8	0.9	0.8	0.7	0.7
mitogen-activated protein kinase 12	MAPK12	0.7	0.7	0.9	0.9	0.8
meningioma (disrupted in balanced translocation) 1	MN1	0.6	0.6	0.8	0.8	0.5
N-acetyltransferase 6	NAT6	0.6	0.7	0.8	0.9	0.7
NIMA (never in mitosis gene a)- related kinase 9	NEK9	0.7	0.7	0.8	0.7	0.9
par-6 partitioning defective 6 homolog alpha	PARD6A	0.5	0.5	0.7	0.8	0.7
RAD52 homolog	RAD52	0.6	0.6	0.8	0.8	0.7
ras homolog gene family, member B	RHOB	0.7	0.6	0.8	0.7	0.7
reprimo, TP53 dependent G2 arrest mediator candidate	RPRM	0.6	0.6	0.8	0.8	0.6
septin 6	septin 6	1.0	1.0	1.0	0.9	0.7
sirtuin 2	SIRT2	0.6	0.6	0.7	0.7	0.9
tumor necrosis factor (ligand) superfamily, member 15	TNFSF15	0.6	0.7	0.8	1.4	0.3
tumor protein p53	TP53	0.8	0.8	0.8	0.7	0.9
tumor protein p73	TP73	0.6	0.7	1.0	0.8	0.5

*^1^Expression level expressed as fold differences of control (control  = 1.0)*.

*^2^2f-O: 2f-LIGRLO-NH_2_ 1 µM* .

*^3^Trypsin: Human Trypsin 50 nM*.

**Table 3:** Cell cycle genes up-regulated^1^ through PAR2 activation by either 2f-LIGRLO-NH_2_ or Trypsin.

Name	Gene	2f-O^2^ 1.5h	2f-O 3h	2f-O 6h	2f-O 12h	Trypsin^3^ 6h
ARP1 actin-related protein 1 homolog A, centractin alpha	ACTR1A	1.2	1.1	1.1	1.2	1.4
cyclin A1	CCNA1	1.3	1.9	1.7	1.3	2.1
cyclin Y-like 1	CCNYL1	1.2	1.3	1.1	1.1	1.5
cell division cycle 123 homolog	CDC123	1.1	1.2	1.1	1.3	1.5
cell division cycle 25 homolog A	CDC25A	2.0	1.7	1.4	1.1	1.0
dual specificity phosphatase 1	DUSP1	1.4	1.6	1.3	1.1	1.3
dual specificity phosphatase 6	DUSP6	2.3	2.6	2.0	1.4	7.5
fumarate hydratase	FH	1.1	1.2	1.2	2.0	1.7
FBJ murine osteosarcoma viral oncogene homolog B	FOSB	1.6	1.3	1.0	1.0	1.1
growth arrest and DNA-damage-inducible, alpha	GADD45A	1.4	1.3	1.1	1.1	1.4
general transcription factor IIH, polypeptide 1	GTF2H1	1.7	1.7	1.3	1.3	1.1
interleukin 8	IL8	2.4	1.9	1.3	2.8	4.5
v-Ki-ras2 Kirsten rat sarcoma viral oncogene homolog	KRAS	1.9	2.1	1.3	1.4	1.2
nucleolar and coiled-body phosphoprotein 1	NOLC1	1.9	1.8	1.7	1.6	1.3
oxidative stress induced growth inhibitor family member 2	OSGIN2	1.1	1.3	1.3	1.2	1.5
PCTAIRE protein kinase 2	PCTK2	1.2	2.0	1.0	1.3	1.4
protein phosphatase 2 (formerly 2A), alpha isoform	PPP2CA	1.3	1.5	1.2	1.2	1.2
protein phosphatase 2 (formerly 2A), beta isoform	PPP2CB	1.2	1.5	1.2	1.3	1.3
protein tyrosine phosphatase type IVA, member 1	PTP4A1	1.5	1.6	1.2	1.1	1.3
SERTA domain containing 1	SERTAD1	2.3	1.9	1.3	1.2	2.0
seven in absentia homolog 1	SIAH1	1.2	1.7	1.0	1.2	1.3
structural maintenance of chromosomes 3	SMC3	1.5	1.7	1.3	1.2	1.0
TAF1 RNA polymerase II	TAF1	1.4	1.4	1.2	1.2	1.1
transcription factor Dp-1	TFDP1	1.1	1.2	1.3	1.4	1.2
topoisomerase (DNA) III alpha	TOP3A	2.3	1.5	1.4	1.2	1.2
U2AF homology motif (UHM) kinase 1	UHMK1	1.2	1.4	1.1	1.2	1.5

*^1^Expression level expressed as fold differences of control (control  = 1.0)*.

*^2^2f-O: 2f-LIGRLO-NH_2_ 1 µM* .

*^3^Trypsin: Human Trypsin 50 nM*.

This data suggests that PAR2 regulates the cell cycle at the transcriptional level, modulating the expression of a number of important genes including crucial regulators of cell cycle transitions such as the cyclin family (CCNA1, CCNF, CCNYL1) and cyclin-dependent kinases (CDK5, CDK10). CCNA1 and CCNYL1 were upregulated 2 and 1.5 fold respectively, and expression of CCNF, CDK5 and CDK10 were suppressed, up to 2 fold for CDK10. Among other key players in cell cycle progression that were affected by PAR2 activation were tumor suppressor p53, cell division cycle members CDC123, CDC20 and CDC25A, and cell cycle regulators such as PPP2CA and PPP2CB.

**Figure 2:**
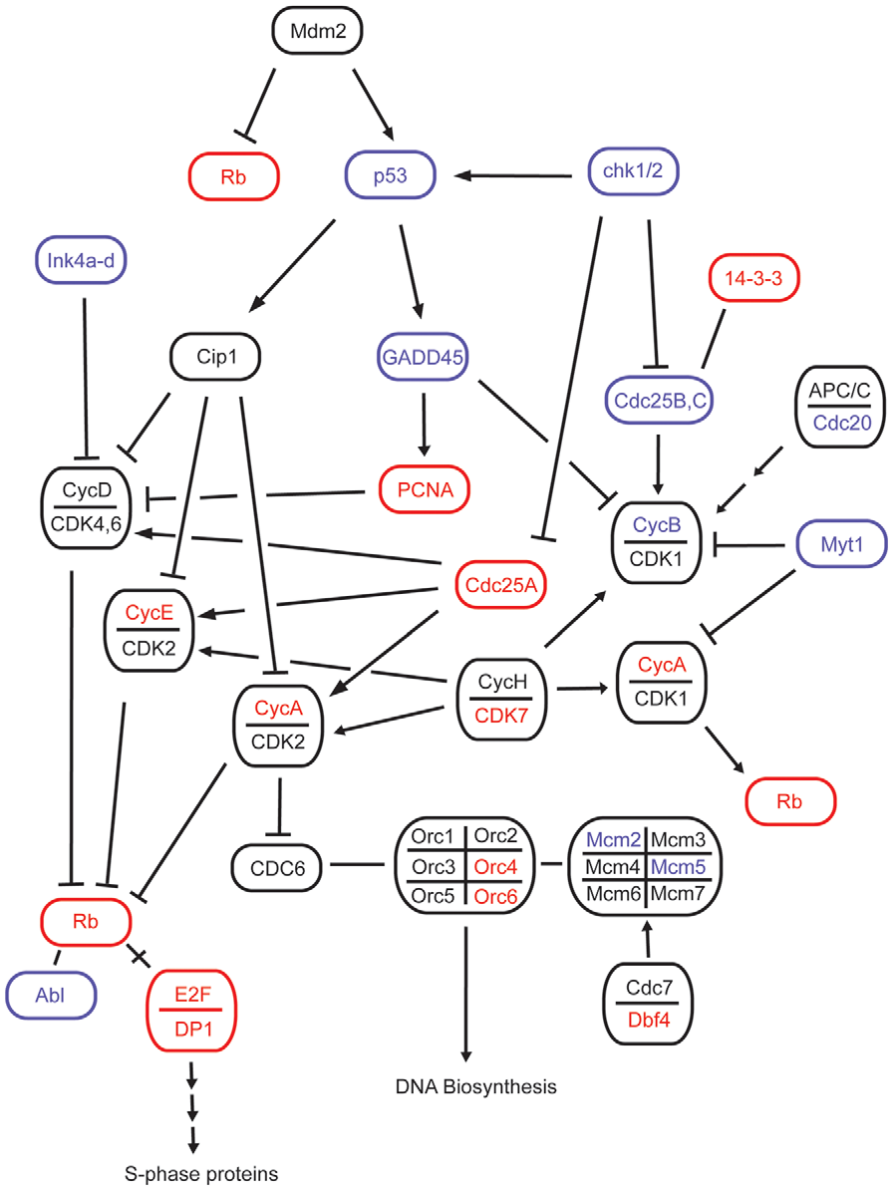
Cell cycle genes regulated by PAR2 (red, up-regulated; blue, down-regulated; black, other related genes). Among key cell cycle components modulated were p53, the cyclin family, and the cdk family. The influence on cell cycle progression is not clear despite reports of PAR2 in cell proliferation.

Surprisingly, not all genes associated with strong proliferative responses were up-regulated following PAR2 activation. Instead, some genes important for cell proliferation were down-regulated, while others were up-regulated (Figure 2). For example, expression of cyclin F and ERK1 were down-regulated ∼2 fold (∼0.5 fold of control), while cyclin A1 and KRAS were up-regulated ∼2 fold. Similar effects were observed for growth suppressing genes NEK9 (suppressed), p53 (suppressed) and CDC123 (induced).

### MAPK genes

An unexpected effect of PAR2 activation by both trypsin and agonist peptide was seen on Mitogen-Activated Protein Kinase (MAPK) genes. PAR2 has been frequently reported to signal through the MAPK pathway but, in our study, ERK1 and MAPK12 were both down-regulated up to 1.4 fold. In addition, 3 members of the dual specificity phosphatase family (DUSP1, 4, 6), which negatively regulate members of the MAP kinase superfamily, were greatly amplified by both trypsin and the agonist peptide. DUSP6 was up-regulated 7 fold by trypsin 6 h post-treatment, compare to 2.5 fold by PAR2 agonist. Genes in the MAP kinase pathway that were most affected by PAR2 activation are shown in Table 4.

**Table 4:** MAPK genes regulated via PAR2 activation by either 2f-LIGRLO-NH_2_ or Trypsin.

Name	Gene	2f-O^2^ 1.5h	2f-O 3h	2f-O 6h	2f-O 12h	Trypsin^3^ 6h
*Down-Regulation*						
adrenergic, alpha-2C-, receptor	ADRA2C	0.5^1^	0.6	0.7	0.6	0.5
CD81 molecule	CD81	0.8	0.7	1.0	0.8	0.8
dual specificity phosphatase 10	DUSP10	0.8	0.8	0.8	0.7	0.9
mitogen-activated protein kinase 3	ERK1	0.7	0.7	0.8	0.7	0.9
Mitogen-activated protein kinase 12	MAPK12	0.7	0.7	0.9	0.9	0.8
fibroblast growth factor receptor 3	FGFR3	0.8	0.8	0.8	0.9	0.9
growth arrest and DNA-damage-inducible, gamma	GADD45G	0.6	0.5	0.8	0.8	0.7
G protein pathway suppressor 1	GPS1	0.8	0.8	0.9	0.8	0.8
G protein pathway suppressor 2	GPS2	0.6	0.5	0.8	0.6	0.9
neurturin	NRTN	0.6	0.7	0.7	0.7	0.8
*Up-Regulation*						
regulator of G-protein signalling 4	RGS4	1.5	1.2	1.3	1.1	2.2
dual specificity phosphatase 1	DUSP1	1.4	1.6	1.3	1.1	1.3
dual specificity phosphatase 4	DUSP4	1.2	1.1	1.1	1.3	1.3
dual specificity phosphatase 6	DUSP6	2.3	2.6	2.0	1.4	7.5
mitogen-activated protein kinase kinase 4	MAP2K4	1.2	1.4	1.1	1.1	1.4
MAP3K12 binding inhibitory protein 1	MBIP	1.1	1.3	1.0	1.1	1.2
p21/Cdc42/Rac1-activated kinase 1	PAK1	1.6	1.5	1.4	1.4	1.3
protein phosphatase 2 (formerly 2A), alpha isoform	PPP2CA	1.3	1.5	1.2	1.2	1.2
protein phosphatase 2 (formerly 2A), beta isoform	PPP2CB	1.2	1.5	1.2	1.3	1.3

*^1^Expression level expressed as fold differences of control (control  = 1.0)*.

*^2^2f-O: 2f-LIGRLO-NH_2_ 1 µM*.

*^3^Trypsin: Human Trypsin 50 nM*.

### Inflammatory mediators

**Figure 3:**
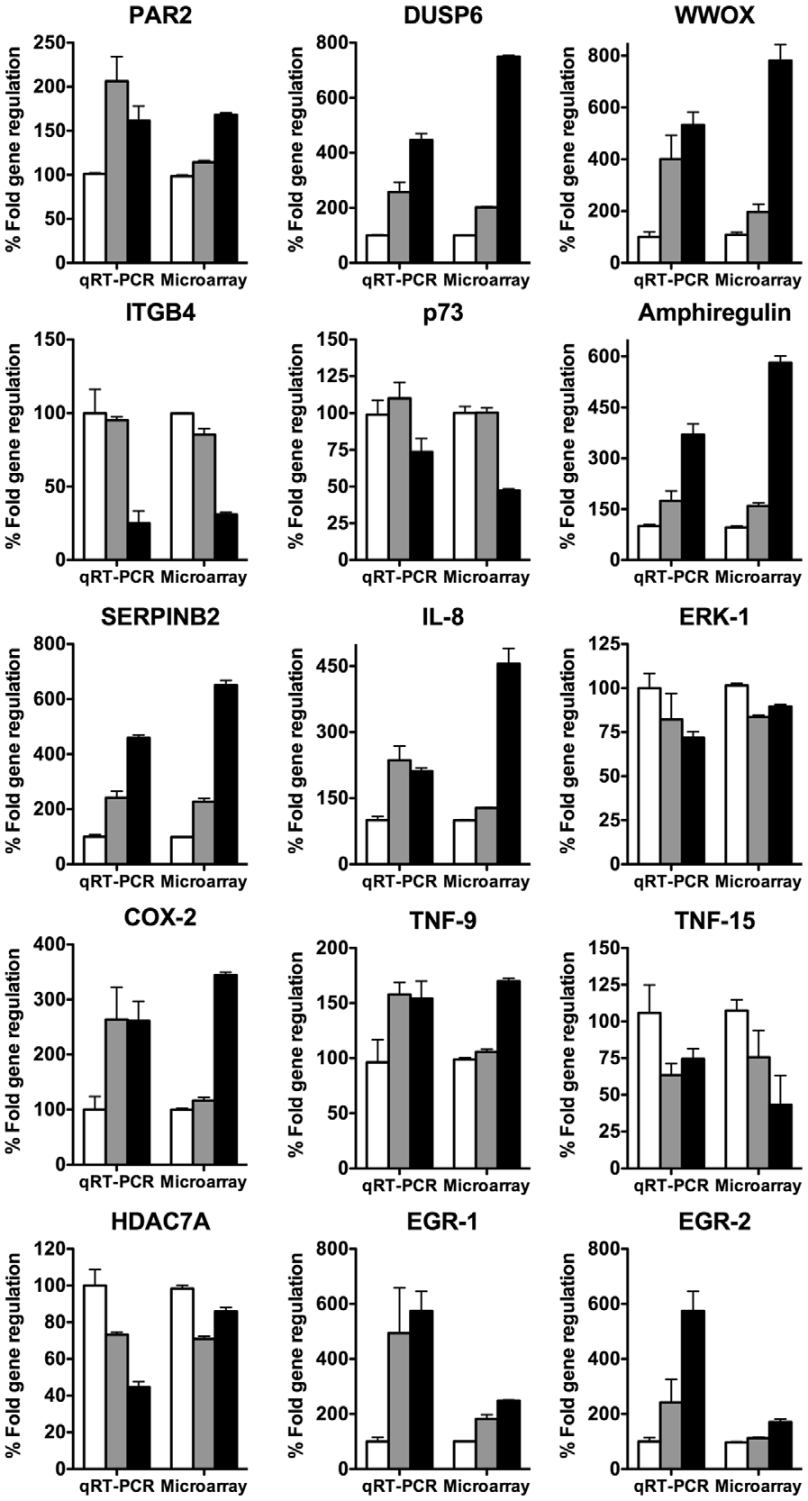
Correlation between microarray gene expression and qRT-PCR. HEK293 cells were treated with trypsin or 2f-LIGRLO-NH_2_ and total RNA was extracted 6 h later. Data represent the average of 3 independent experiments. Columns (negative control, white; 2f-LIGRLO-NH_2_, grey; trypsin, black) show representative regulation of PAR2, DUSP6, WWOX, ITGB4, p73, Amphiregulin, SERPINB2, Il-8 precursor, ERK-1, COX-2, TNF-9, TNF-15, HDAC-7A, EGR-1 and EGR-2 by PAR2 activation. Results from qRT-PCR were compared to microarray data at equal times.

The important inflammatory mediator cyclooxygenase-2 (COX-2) has recently been reported to be activated with induced PAR2 expression and activation in gastric epithelial cells [41]. In our array data, COX-2 was up-regulated 3.5 and 3 fold by trypsin and PAR2 agonist peptide respectively (Table 5). Up-regulation of COX-2 and other inflammatory mediators was verified by quantitative real-time PCR (Figure 3). Other genes up-regulated by PAR2 activation included interleukin 1 family member 9 (1.7 fold), interleukin 8 (4.5 fold) and its receptor (1.2 fold), coagulation factor VIII (1.5 fold) and TNF-9 (1.7 fold). Interestingly, TNF-15 was instead reduced to 0.3 fold of untreated control.

**Table 5:** PAR2 activated genes associated with inflammation induced by either 2f-LIGRLO-NH_2_ or Trypsin.

Name	Gene	2f-O^2^ 1.5h	2f-O 3h	2f-O 6h	2f-O 12h	Trypsin^3^ 6h
*Genes involved in Inflammatory and Immune response*						
prostaglandin-endoperoxide synthase 2	COX-2	3.1^1^	1.9	1.2	1.2	3.4
Interleukin 8 Precusor	IL-8	2.4	1.9	1.3	2.8	4.5
Interleukin 1 family, member 9	IL1F9	1.4	1.2	1.2	1.1	1.8
Interleukin 8 Receptor	IL8R	1.0	1.3	1.1	1.2	1.2
fibrinogen gamma chain	FGG	1.8	1.5	1.5	1.4	1.2
coagulation factor VIII	F8	1.5	1.2	1.0	1.0	1.4
TNF superfamily, member 9	TNF9	1.4	1.2	1.1	1.1	1.7
*Complement Pathway*						
CD55 molecule	CD55	1.2	1.3	1.0	1.0	1.3
complement component 1, q subcomponent like	C1QL1	0.7	0.7	0.8	0.8	1.0
complement component 1, r subcomponent	C1R	0.8	0.8	0.8	0.7	0.9
complement component 4B	C4B	0.7	0.7	0.7	0.7	0.7
complement factor D	CFD	0.8	0.9	0.9	1.0	0.8

*^1^Expression level expressed as fold differences of control (control  = 1.0)*.

*^2^2f-O: 2f-LIGRLO-NH_2_ 1 µM*.

*^3^Trypsin: Human Trypsin 50 nM*.

### Complement genes

**Figure 4:**
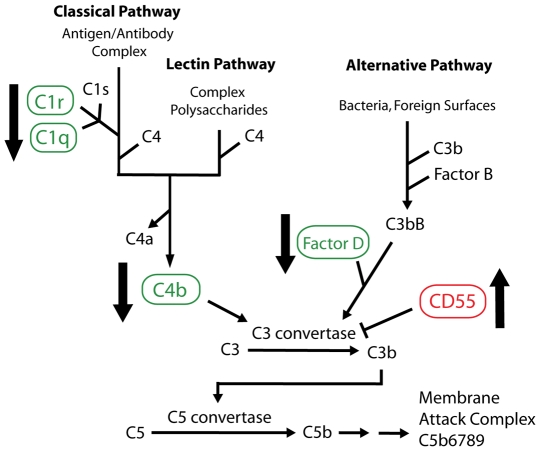
Effects of PAR2 activation on complement pathways. Relationship between genes regulated by PAR2 (black arrows) and classical, alternative and lectin pathways of complement activation. Five key genes regulated by PAR2 activation were C1q, C1r, C4b, factor D and CD55 (decay accelerating factor). C1r/q subunits of C1 are in the classical pathway; C4b is essential for formation of C3 convertase in the classical and lectin pathways; Factor D is similarly important for C3 convertase in the alternative pathway; CD55 actively breaks down C3 convertase contributing to anti-complement effects of PAR2. Such regulation of all these genes will limit formation of pro-inflammatory anaphylatoxins (C3a, C5a) and membrane attack complex.

Five genes associated with the complement pathway were conspicuously though subtly regulated by both trypsin and PAR2 agonist peptide (Table 5). Two subunits of C1 complex, C1R and C1QL1, were showing expression ∼0.7 fold of control. Expression of C4b, which forms C3 convertase from classical and lectin pathways, was also ∼0.7 fold of control after 12 h. In addition, a protein responsible for C3 convertase production from the alternative pathway, factor D, was slightly suppressed (0.8 fold of control). On the other hand, expression of decay accelerating factor (CD55) increased 1.3 times as a result of trypsin treatment. Together, this data suggests that PAR2 is upstream of complement activation and is consistent with PAR2 activation limiting complement activation (Figure 4).

### HDAC/sirtuin family

Array data showed that the expression of a number of histone deacetylase (HDAC) genes (HDAC3, 7A, 11) was suppressed by PAR2 activation, as were the related sirtuins 2, 3, 5 and 6 (Table 6). Interestingly, sirtuin 1 was the only member of the HDAC/sirtuin family that was detectably up-regulated by both trypsin (1.3 times) and PAR2 agonist peptide (1.4 times). Moreover, the reduction in expression of all three HDACs and sirtuin 2 was continuous for up to 12 h after treatment, whereas the other sirtuin members slowly returned to normal level over 12 h.

**Table 6:** Transcriptional regulation^1^ of HDAC and sirtuin enzymes by PAR2 activation via either 2f-LIGRLO-NH_2_ or Trypsin.

Gene Name	2f-O^2^ 1.5h	2f-O 3h	2f-O 6h	2f-O 12h	Trypsin^3^ 6h
Histone Acetyltransferase-1	1.1	1.2	1.1	1.3	1.2
*HDAC family*					
histone deacetylase 1	1.0	1.0	0.9	0.9	0.9
histone deacetylase 2	1.0	1.1	1.0	0.9	1.1
histone deacetylase 3	0.7	0.6	0.8	0.7	0.8
histone deacetylase 4	0.8	0.6	0.8	0.6	1.3
histone deacetylase 5	0.8	0.9	0.9	0.9	0.9
histone deacetylase 6	0.9	0.9	0.8	1.0	0.9
histone deacetylase 7A	0.5	0.4	0.7	0.6	0.9
histone deacetylase 8	1.3	1.2	1.2	1.2	0.9
histone deacetylase 9	1.1	1.1	0.8	1.2	1.5
histone deacetylase 11	0.6	0.6	0.8	0.7	0.6
*Sirtuin family*					
sirtuin 1	1.3	1.4	1.0	1.0	1.3
sirtuin 2	0.6	0.6	0.7	0.7	0.9
sirtuin 3	0.8	0.7	0.9	0.8	0.6
sirtuin 4	0.9	0.9	1.0	1.0	1.1
sirtuin 5	0.9	0.9	0.9	1.0	0.8
sirtuin 6	0.8	0.8	1.0	1.0	0.8
sirtuin 7	1.0	0.9	0.9	0.9	0.9

*^1^Expression level expressed as fold differences of control (control  = 1.0)*.

*^2^Peptide: 2f-LIGRLO-NH_2_ 1 µM*.

*^3^Trypsin: Human Trypsin 50 nM*.

### Metabolism

Of ∼2500 genes regulated similarly by trypsin and PAR2 agonist peptide, more than 1000 genes are reportedly involved in different aspects of cell metabolism, including biopolymer metabolism (471 genes), macromolecular metabolism (687 genes) and primary metabolism (1023 genes), with the highest number of genes involved and the lowest p-value. Some 44 genes showing >2 fold changes are listed in Table 7 and 8. Overall, changes in expression levels were greater for metabolism genes than for genes in other pathways, with prolonged effects lasting up to 12 h. Genes encoding early growth response 1 and 2 proteins were up-regulated by PAR2 activation (EGR1 2.8x, EGR2 2x), so were the EGR binding proteins NAB1 (2 fold) and NAB2 (4 fold). Other notable genes down-regulated by trypsin were cathepsin D (0.3 fold of control) and telomerase reverse transcriptase (0.5 fold of control), while protein phosphatase 2C was upregulated (3 fold) by PAR2 activating peptide.

**Table 7:** PAR2 activated genes involved in metabolism down-regulated by either 2f-LIGRLO-NH_2_ or Trypsin.

Name	Gene	2f-O^2^ 1.5h	2f-O 3h	2f-O 6h	2f-O 12h	Trypsin^3^ 6h
ATP-binding cassette, sub-family A, member 2	ABCA2	0.5	0.5	0.7	0.6	0.6
Aldehyde dehydrogenase 1 family, member A3	ALDH1A3	0.8	0.8	0.6	0.7	0.4
ATPase, Ca++ transporting, ubiquitous	ATP2A3	0.6	0.7	0.7	0.7	0.5
Cyclin-dependent kinase (CDC2-like) 10	CDK10	0.5	0.4	0.7	0.6	0.8
Cathepsin D	CTSD	0.7	0.6	0.9	0.7	0.3
2,4-dienoyl CoA reductase 2, peroxisomal	DECR2	0.6	0.5	0.7	0.6	0.9
EPH receptor B4	EPHB4	0.6	0.5	0.8	0.6	0.8
Epoxide hydrolase 2, cytoplasmic	EPHX2	0.6	0.6	0.7	0.6	0.5
Histone deacetylase 7A	HDAC7A	0.5	0.4	0.7	0.6	0.9
3-hydroxymethyl-3-methylglutaryl-Coenzyme A lyase	HMGCL	0.6	0.5	0.7	0.7	0.8
Hydroxy-delta-5-steroid dehydrogenase, 3 beta- and steroid delta-isomerase 7	HSD3B7	0.7	0.6	0.7	0.7	0.5
Inositol polyphosphate-5-phosphatase	INPP5D	0.6	0.7	0.6	0.6	0.5
PCTAIRE protein kinase 3	PCTK3	0.5	0.4	0.7	0.6	0.8
Protein disulfide isomerase family A, member 2	PDIA2	0.6	0.7	0.7	0.8	0.5
Protein tyrosine phosphatase, non-receptor type 6	PTPN6	0.4	0.4	0.5	0.5	0.8
Solute carrier family 7 (cationic amino acid transporter, y+ system), member 8	SLC7A8	0.8	0.7	0.8	0.7	0.4
SMAD family member 6	SMAD6	0.6	0.6	0.7	0.8	0.5
Telomerase reverse transcriptase	TERT	0.7	0.8	0.8	1.0	0.5
Tyrosine kinase, non-receptor, 2	TNK2	0.6	0.4	0.6	0.6	0.8
Tumor protein p73	TP73	0.6	0.7	1.0	0.8	0.5
TSC22 domain family, member 3	TSC22D3	0.6	0.6	0.6	0.7	0.4
Zinc finger and BTB domain containing 16	ZBTB16	0.6	0.5	0.7	0.8	0.8

*^1^Expression level expressed as fold differences of control (control  = 1.0)*.

*^2^2f-O: 2f-LIGRLO-NH_2_ 1 µM*.

*^3^Trypsin: Human Trypsin 50 nM*.

**Table 8:** PAR2 activated genes involved in metabolism up-regulated by either 2f-LIGRLO-NH_2_ or Trypsin.

Name	Gene	2f-O^2^ 1.5h	2f-O 3h	2f-O 6h	2f-O 12h	Trypsin^3^ 6h
Acyl-CoA synthetase long-chain family member 4	ACSL4	2.0^1^	2.1	1.5	1.4	1.6
Catenin (cadherin-associated protein), beta 1	CTNNB1	2.2	1.5	1.4	1.2	1.5
Discs, large homolog 1	DLG1	2.4	1.4	1.3	1.1	1.5
Dual specificity phosphatase 6	DUSP6	2.3	2.6	2.0	1.4	7.5
Early growth response 1	EGR1	2.8	2.0	1.8	1.1	2.5
Early growth response 2	EGR2	2.1	1.5	1.1	1.0	1.7
Ets variant gene 4 (E1A enhancer binding protein, E1AF)	ETV4	1.3	1.4	2.0	1.3	2.7
Exosome component 3	EXOSC4	1.3	2.1	1.3	1.2	1.9
3-hydroxy-3-methylglutaryl-Coenzyme A synthase 1 (soluble)	HMGCS1	1.6	1.8	1.5	2.1	1.9
Homer homolog 1	HOMER1	2.4	2.2	1.0	0.9	1.7
Inhibitor of DNA binding 4, dominant negative helix-loop-helix protein	ID4	1.5	1.6	1.2	1.4	2.6
Kruppel-like factor 5 (intestinal)	KLF5	2.2	2.1	1.5	1.3	1.6
Myeloid/lymphoid or mixed-lineage leukemia (trithorax homolog, Drosophila)	MLLT	2.0	1.8	1.1	1.4	1.2
Matrix metallopeptidase 3	MMP3	1.3	1.5	1.5	1.0	2.2
NGFI-A binding protein 1 (EGR1 binding protein 1)	NAB1	2.1	2.2	1.2	1.3	1.4
NGFI-A binding protein 2 (EGR1 binding protein 2)	NAB2	4.0	2.3	2.5	1.4	2.1
Oligodendrocyte lineage transcription factor 2	OLIG2	2.2	2.4	1.4	1.2	1.7
Protein phosphatase 2C, magnesium-dependent, catalytic subunit	PPM2C	2.9	1.9	1.5	1.4	1.3
prostaglandin-endoperoxide synthase 2 (COX-2)	PTGS2	3.1	1.9	1.2	1.2	3.4
Protein tyrosine phosphatase, non-receptor type 14	PTPN14	2.2	2.3	2.0	2.1	1.4
Topoisomerase (DNA) III alpha	TOP3A	2.3	1.5	1.4	1.2	1.2
Zinc finger and BTB domain containing 43	ZBTB43	2.4	1.9	1.4	1.2	1.2

*^1^Expression level expressed as fold differences of control (control  = 1.0)*.

*^2^2f-O: 2f-LIGRLO-NH_2_ 1 µM*.

*^3^Trypsin: Human Trypsin 50 nM*.

### Genes regulated by PAR2 vs. PAR1

Modulating effects of thrombin have been reported on a small set of genes by PAR1 activation [40]. Of those ∼11,000 genes, at least 65 were induced by thrombin. We compared those 65 genes with genes regulated by PAR2 from our study and found 4 genes that showed the opposite trend to that reported following PAR1 activation. A further 14 genes were regulated in a similar fashion by both PAR2 agonists (Table 9). Notably, chemokine (C-C motif) ligand 2 expression was amplified 4 fold after treatment by either thrombin or trypsin. Thrombospondin-1 was previously only known to be regulated through PAR1, but our data shows that PAR2 can also up-regulate it at a comparable level (2.5 vs 2.2 fold).

**Table 9:** Genes regulated by both PAR1 and PAR2 activation.

		PAR1^2^			PAR2	2f-O^3^			Trypsin^4^
Name	Gene	1.5h	6h	12h	1.5h	3h	6h	12h	6h
*Opposite Regulation*									
retinol dehydrogenase 5	RDH1	1.5^1^	1.4	1.6	0.8	0.9	0.8	0.9	0.8
cyclin D2	CCND2	1.4	2.4	2.5	0.9	0.7	0.8	0.8	0.8
smoothelin	SMTN	1.1	1.5	1.6	0.7	0.6	0.9	0.7	0.6
ras homolog gene family, member B	RHOB	1.5	1.9	1.4	0.7	0.6	0.8	0.7	0.7
*Similar Regulation*									
monoglyceride lipase	MGLL	1.2	1.6	1.9	1.0	1.2	1.0	1.1	1.2
uridine-cytidine kinase 2	UMPK	1.2	1.5	1.7	1.1	1.2	1.2	1.2	1.3
CD44 molecule	CD44	1.1	1.0	1.7	1.6	1.3	1.1	1.1	1.3
FOS-like antigen 1	FOSL1	1.8	1.7	1.4	1.2	1.2	1.2	1.1	2.0
TNF receptor superfamily, member 12a	TNFRSF12A	2.1	2.1	2.1	1.2	1.1	1.2	1.1	1.3
RAB3A, member RAS oncogene family	RAB3A	1.9	1.7	1.4	1.1	1.2	1.0	0.8	1.3
Kruppel-like factor 6	COPEB	1.6	1.6	1.3	1.1	1.2	1.1	1.2	1.1
chemokine (C-C motif) ligand 2	CCL2	2.7	4.0	2.1	1.7	1.8	1.2	1.4	4.2
gremlin 1	CKTSF1B1	1.2	2.1	2.1	1.3	1.5	1.2	1.1	2.8
coronin, actin binding protein, 1C	CORO1C	1.2	2.0	1.8	1.2	1.1	1.1	0.9	1.5
thrombospondin 1	THBS1	1.2	2.5	1.9	2.1	2.2	1.3	1.2	1.6
syndecan 4	SDC4	2.0	1.4	1.5	1.1	1.1	1.2	1.1	2.3
immediate early response 3	IER3	2.4	1.8	1.8	1.2	1.0	1.1	1.1	1.4
connective tissue growth factor	CTFG	2.4	1.9	1.5	1.6	1.3	1.2	1.1	1.9

*^1^Expression level expressed as fold differences of control (control  = 1.0).*

*^2^PAR1 results extracted from McLaughlin et.al. (2005) J.Biol.Chem.280:22172-22180.*

*^3^PAR2 2f-O: 2f-LIGRLO-NH_2_ 1 µM.*

*^4^Trypsin: Human Trypsin 50 nM.*

## Discussion

The distinctive mode of endogenous PAR2 activation, with no highly specific endogenous ligand known, makes it difficult to clarify specific functions for PAR2. In this study, we used a novel approach to identify for the first time the specific gene expression profile induced by PAR2 activation. By comparing the gene expression separately induced by trypsin and a PAR2 agonist peptide, and identifying intersecting gene sets similarly up- or down- regulated following both treatments, we can be more confident about the effects of specific PAR2 modulation than if either activation method was used alone.

### PAR2 activation induces PAR2 expression

PAR2 expression is up-regulated following PAR2 activation. This is logical for PAR2, as endogenous activators for the receptor are serine proteases, which irreversibly activate PAR2 through N-terminal cleavage. New PAR2 must therefore be produced in the cell and expressed on the cell surface to account for the observed up-regulation of PAR2 mRNA in response to the two extracellular PAR2 agonists.

### WWOX, p73 and ITGB4

WWOX is a tumor suppressor gene [42] that shows reduced or no expression in certain cancer models, while over-expression induces dramatic inhibition of tumorigenicity in breast cancer. Knock out mice develop osterosarcoma and die within 4 weeks. WWOX associates with some proteins through its WW-domain, attenuating translocation into the nucleus and subsequent transcriptional activity. For example, p73 expression [43] can be tumor suppressive or oncogenic depending on isoforms of its gene product [44]. p73 expression was downregulated herein by PAR-2 activation, along with 8 fold increases in WWOX, suggesting that PAR-2 leads to reduced p73 transcriptional activity [43]. Further research into effects of PAR-2 on WWOX regulation in cancer could also be interesting as the tumor suppressor gene is up-regulated by PAR-2, even though PAR-2 has been reported to induce proliferation in certain carcinomas.

Integrin beta 4 (ITGB4) is a laminin receptor that is upregulated in tumor cells and angiogenic endothelial cells [45]. Under normal conditions, this protein regulates cellular migration and proliferation but can promote invasive growth when coupled to oncogenic receptor tyrosine kinases. Down-regulation by PAR2 indicates a self-regulatory effect in HEK293 cells, in line with results for the WWOX gene. It is possible that the proliferative effect of PAR2 is countered via regulating other genes.

### AREG

The product of this gene, amphiregulin, is a member of the epidermal growth factor family [46]. Amphiregulin is activated by metalloproteases and can be induced by cAMP activation, and increased levels of amphiregulin expression and release in airways, after activation by human airway trypsin-like protease through PAR2 receptor activation, result in mucus hypersecretion in chronic obstructive airway disease [46]. Our array data has demonstrated that amphiregulin expression is also substantially induced in kidney cells by PAR2 activation and this may be important in proliferation of kidney cells.

### Cell cycle progression

PAR2 has been associated with cell proliferation in cancer and metastasis [6], [7], [8], [9]. Our finding of differential up- and down-regulation of genes associated with the cell cycle was surprising to us, and contrary to an expectation of up-regulation of genes involved in cell proliferation. The implications are not yet clear, except that there would seem to be no single message for, or against, proliferation. Thus, the precise role of PAR2 activation in proliferation at least in HEK293 cells remains obscure based on microarray data alone.

### MAPK pathway

PAR2 activation also unexpectedly down-regulated expression of MAPK associated genes, including ERK-1. PAR2 is known to couple to Gi, and both ERK and MEK are signal transducers in the Gi signaling cascade. There was also a strong (7-fold) upregulation of DUSP6, a negative regulator for the MAPK superfamily, after treatment with trypsin. Other DUSP family members affected included DUSP1, 4 and 10. DUSP4 specifically targets JNK, and both DUSP1 and 10 have demonstrated roles in inflammation. To our knowledge, PAR2 has not previously been reported to negatively regulate the MAPK pathway.

### Complement regulation

Another surprise was that PAR2 activation led to some differential expression of 5 key genes associated with complement activation (Figure 4). Complement genes 1r and 4a, which are particularly important for complement activation via the classical pathway, were slightly down-regulated, suggesting that PAR2 activation indirectly leads to inhibition of the classical pathway of complement activation. Similarly, factor D which is crucial for forming the C3 convertase that is pivotal for complement activation by classical, lectin and alternative pathways, was also slightly down-regulated (0.8 fold of control) by PAR2 activation. Interestingly, the decay accelerating factor (CD55) which has the reverse effect in complement pathways was up-regulated by PAR2. Together these findings support anti-complement properties for PAR2, consistent with an anti-inflammatory role at least for the kidney cells studied here. Also, as a consequence of acting as an upstream negative regulator of complement activation, PAR2 activation is expected to reduce formation of the pro-inflammatory anaphylatoxins (C3a, C4a, C5a) as well as membrane attack complex (MAC) required for host-mediated destruction of infectious organisms.

### Other inflammatory mediators

PAR2 activation reportedly displays both pro- [14], [17] and anti- inflammatory [10], [12], [13], [15], [16] responses in different disease models, including asthma, arthritis, and irritable bowel syndrome. These seemingly paradoxical roles for PAR2 in inflammation have been based mainly on observations of PAR2-activating peptides with low potency and uncertain receptor selectivity, and so there remains doubt about whether both pro- and anti- inflammatory properties can truly be ascribed to PAR2 activation. To date, the mechanisms that PAR2 uses to mediate these inflammatory responses have not been fully elucidated, but if PAR2 really is both pro- and anti- inflammatory these properties may be highly context and cell dependent.

Cyclooxygenase-2 expression was found here to be significantly amplified by PAR2 activation. COX-2 is an important downstream pro-inflammatory mediator. Up-regulation of this and other inflammatory mediators (e.g. IL-8 and receptor, IL-1, TNF-9) is consistent with a pro-inflammatory role for PAR2 activation, in contrast to the anti-inflammatory complement effects above. This is the first time at the transcriptional level that PAR2 has been shown to trigger both pro- and anti- inflammatory responses in the one cell line and is consistent with the notion that PAR2 activation could be pro- or anti- inflammatory depending upon circumstances. Which physiological response is exhibited may indeed be a function of cell/tissue type and the environmental concentrations of receptors/inhibitors capable of masking inflammatory responses.

Histone deacetylases (HDACs) and their inhibitors have been associated with both pro- and anti-inflammatory roles and are being increasingly associated with inflammation [47], [48]. Inhibitors have been found to exhibit potential therapeutic roles in rat adjuvant- [49] and mouse antibody- [50] induced arthritis, mouse inflammatory renal disease [51], airways inflammation in mice [52], experimental autoimmune encephalomyelitis [53], and COPD [54]. Up-regulation of HDACs by lipopolysaccharide (LPS) or use of some HDAC inhibitors (particularly inhibitors of class I HDACs) results in amplified expression of proinflammatory genes such as COX-2 [55]. This is particularly relevant to, and consistent with, findings in the present study where PAR2 activation leads to up-regulation of certain class I HDACs (HDAC 3, 11) and class II HDACS (HDAC 7), as well as up-regulation of pro-inflammatory genes such as COX-2.

### Metabolism

There is almost no literature on PAR2 activation and metabolism, yet the results herein showed that ∼50% of genes regulated by PAR2 activation in kidney cells are involved in metabolism. The significance of this new observation for PAR2 activation in terms of cellular and physiological mechanisms remains to be elucidated, but points to the need for studies that investigate PAR2 regulation in both normal metabolism and metabolic dysfunction.

### PAR2 versus PAR1

PAR1 is activated in a similar manner to PAR2 by proteases (thrombin, other coagulation proteases, different activating hexapeptides). It has been closely linked to the coagulation cascade and platelet activation, which is critical for hemostasis and thrombosis. PAR1 was recently implicated in tumor progression, especially cancer metastasis and angiogenesis [4]. This is consistent with findings in the present microarray study, where 14 genes that were up-regulated by PAR1 [40] were also up-regulated by PAR2. Of these, 9 genes are involved in regulation of tumorigenesis, a finding which suggests that PAR1 and PAR2 may share some common key signaling pathways in cancer. In addition, thrombospondin-1 is up-regulated by PAR2 activation. THBS-1 is a large extracellular matrix glycoprotein shown to cause platelet activation, angiogenesis and wound healing. Previously, it was known to be only activated by PAR1, but now we suggest that PAR2 can also trigger expression of thrombospondin-1.

### Conclusions

The present study provides a valuable and decisive platform upon which to base new hypotheses and pharmacological studies for probing the specific roles for PAR2 activation in physiology and disease. It is clear from this study that PAR2 activation is self-sustaining and strongly implicated in cell metabolism, proliferation and inflammation. The study particular highlights the need for new research on PAR2 in metabolism and metabolic dysfunction. Tumour and growth factor genes like WWOX, p73, ITGB4 and AREG were strongly up-regulated by PAR2 activation, supporting roles for PAR2 in proliferation, tumour growth and, perhaps surprisingly, also tumour suppression. The evidence for anti-inflammatory (anti-complement) and pro-inflammatory (COX2, HDACs, IL8, IL1, TNF, DUSP1, DUSP10, AREG) effects in HEK293 cells is the first evidence at the gene expression level for a dual capacity of PAR2 in regulating inflammatory pathways that may have important implications in the kidney and possibly in kidney-related disease. These observations validate to some extent the seemingly paradoxical literature on possible pro- and anti- inflammatory roles for PAR2, which have been simultaneously witnessed here at the transcriptional level in the one cell line. Finally, some genes were expressed with similar profiles following activation by either PAR1 or PAR2, these mainly being associated with tumorigenesis and cancer progression, implicating a possible sharing of proliferative mechanisms between these two receptors on cell surfaces. This study supports the idea that both PAR2 agonists and antagonists might have valuable therapeutic roles in different tissues and encourages the development of both agonists and especially antagonists that are selective for PAR2 as potential new therapies for cancers and inflammatory conditions.

## Materials and Methods

### Cell Culture

HEK293 cells were maintained in DMEM with 10% fetal bovine serum, penicillin/streptomycin (5000 units/mL; 5000 µg/mL), 1X Non-Essential Amino Acid, and L-glutamine in 95% air/5% CO2 at 37°C. The cells were seeded at 1×105 cells/mL and subcultured after detachment with 0.05% trypsin/0.5 mM EDTA. Cell culture reagents were purchased from Invitrogen, USA.

### HEK293 treatment

Cells were grown to 90% confluence, switched to serum free medium containing 0.03% bovine serum albumin (BSA), and incubated for 3 h. Cells were then treated with either 50 nM trypsin (Invitrogen, USA), 1 µM PAR2 agonist peptide (2f-LIGRLO-NH_2_; synthesized in-house) or vehicles (phosphate buffered saline, pH 7.4) and incubated for additional 90 min, 3, 6 or 12 hours. Cells were then washed three times with cold PBS, ready for RNA extraction.

### RNA extraction and reverse transcription

Total RNA was extracted from cells using an RNeasy mini kit (Qiagen, USA) and following manufacturer's instructions. RNA was checked on an agarose gel for quality and concentration determined by measuring absorbance at λ = 260/280 nm on a spectrophotometer. Total RNA was then reverse transcripted with Superscript III (Invitrogen, USA) using random oligo dT primer. Reagents were incubated 15 min, 25°C; then 50 min, 50°C; 15 min, 70°C.

### Gene Expression Profiling

Arrays were obtained from the SRC Microarray Facility, University of Queensland (ARC Centre for Functional and Applied Genomics) and comprised 18,664 human gene-specific oligo-nucleotides spotted on coated slides. All samples were compared to controls in triplicate, with one of three hybridizations involving a dye reversal. Total RNA underwent one rounds of linear amplification using MessageAmp aRNA Kit (Ambion Inc. TX, USA). For each comparison, 2 µg of amplified RNA was labelled with Cy3-dUTP or Cy5-dUTP (Amersham Biosciences, Australia). Cy3 and Cy5 hybridization signals were collected using the Agilent 600B Array Scanner. Intensity and background data for each element were calculated using Imagene 5.5 (BioDiscovery Inc., Australia).

### Data Analysis

Microarray data were analyzed as described [56]. Briefly, microarray data were imported and the entire dataset is available from NCBI via the GEO database (accession number: GSE24332). Raw data from each hybridization was compiled and subjected to print tip intensity independent Lowess normalization using the R statistical software package data and analyzed using LIMMA (http://bioinf.wehi.edu.au/limma/). This normalization was implemented in BASE using scripts developed by Ola Spjuth of Linnaeus Centre for Bioinformatics (http://www.lcb.uu.se/baseplugins.php). Normalized values were used to calculate B-statistics, which were analyzed with allowance for the correlation between adjacent duplicate spots printed on the same array. Differential expression was defined using a robust statistical method rather than simple fold change. All genes were ranked using B-statistics where both fold change and variance of signals in replicates was used to determine the likelihood that genes were truly differentially expressed. A B-score >0 for a gene translates to a >50% probability of being truly differentially expressed. This analysis was executed using the Bio-conductor package as a plug in tool in BASE. Full documentation of the array fabrication, gene content, experimental procedures and all results are available in accordance with MIAME (http://www.mged.org/ Workgroups/MIAME/miame.html) guidelines at http://kidney.scgap.org/base. Genes similarly regulated by both treatments were annotated and compared using Database for Annotation, Visualisation and Intergrated Discovery (DAVID, http://david.abcc.ncifcrf.gov/).

### Quantitative RT-PCR

Duplicate samples were treated with trypsin (50 nM) or PAR2 agonist (1 µM) for 6 h. Total RNA was extracted and reverse transcripted as outlined. 50 ng of cDNA was incubated with reagents and relevant primers as listed. PAR2: forward primer, 5′ GGG TTT GCC AAG TAA CGG C 3′, reverse primer, 5′ GGG AAC CAG ATG ACA GAG AGG 3′; DUSP6: forward primer, 5′ ATC ACT GGA GCC AAA ACC TG 3′, reverse primer, 5′ CAG CCA AGC AAT GTA CCA AG 3′; WWOX: forward primer, 5′ GAA AAC GAG TGG CAG GAG AT 3′, reverse primer, 5′ GCT GGG TTT ACT ACG CCA AT 3′; ITGB4: forward primer, 5′ GGC AAC CGG GAC TAC ATC C 3′, reverse primer, 5′ CGC AGG AGG GAG TCA ACT T 3′; p73: forward primer, 5′ GTC AAG CCG GGG GAA TAA TGA 3′, reverse primer, 5′ CTC AGC AGA TTG AAC TGG GC 3′; Amphiregulin: forward primer, 5′ GTC CTC GGG AGC CGA CTA T 3′, reverse primer, 5′ GGG GGC TTA ACT ACC TGT TCA A 3′; SERPINB2: forward primer 5′ ATG CAG TTA CCC CCA TGA CT 3′, reverse primer, 5′ CGC ATC AGG ATA ACT ACC CTT C 3′; IL-8 precursor: forward primer, 5′ AAG AAA CCA CCG GAA GGA AC 3′, reverse primer, 5′ AGC TGC AGA AAT CAG GAA GG 3′; ERK-1: forward primer 5′ CTG GAT CAG CTC AAC CAC ATT 3′, reverse primer 3′ AGA GAC TGT AGG TAG TTT CGG G 3′; COX-2: forward primer, 5′ ATA TGT TCT CCT GCC TAC TGG AA 3′, reverse primer, 5′ GCC CTT CAC GTT ATT GCA GAT G 3′; TNF-9: forward primer, 5′ TGG TGG CCC AAA ATG TTC TG 3′, reverse primer, 5′ AGA AGA CAT AGT AGA CTC CAG CC 3′; TNF-15: forward primer, 5′ CTT GCA GGA CTC ACC ACA TAC 3′, reverse primer, 5′ CGT CTG CTC TAA GAG GTG CAT 3′; HDAC7A: forward primer, 5′ AGA GCA AGC GAA GTG CTG TAG 3′, reverse primer, 5′ GGG CTC CAG GGT TCT GTA G 3′; EGR-1: forward primer, 5′ ACC TGA CCG CAG AGT CTT TTC 3′, reverse primer, 5′ GCC AGT ATA GGT GAT GGG GG 3′; EGR-2: forward primer, 5′ GCC AAG GCC GTA GAC AAA ATC 3′, reverse primer, 5′ CCA CTC CGT TCA TCT GGT CA 3′. PCR was run on ABI PRISM 7000 (Applied Biosystem, Australia) with the following cycle conditions: 50°C for 2 min, 95°C for 10 min then 40 cycles of 95°C for 15 s and 60°C for 1 min, completed with 25°C for 2 min. Fluorescence data was collected during the extension phase (60°C). Raw real time data was analyzed using ABI Prism 2000 SDS v2.0 software (Applied Biosystem, Australia).
